# Bottom‐up processes drive reproductive success in an apex predator

**DOI:** 10.1002/ece3.3800

**Published:** 2018-01-11

**Authors:** Joshua H. Schmidt, Carol L. McIntyre, Carl A. Roland, Margaret C. MacCluskie, Melanie J. Flamme

**Affiliations:** ^1^ Central Alaska Network U.S. National Park Service Fairbanks AK USA; ^2^ Denali National Park and Preserve U.S. National Park Service Fairbanks AK USA; ^3^ Yukon‐Charley Rivers Preserve and Gates of the Arctic National Park and Preserve U.S. National Park Service Fairbanks AK USA

**Keywords:** bottom‐up control, golden eagle, population dynamics, predator–prey, snowshoe hare, top‐down control, willow ptarmigan

## Abstract

One of the central goals of the field of population ecology is to identify the drivers of population dynamics, particularly in the context of predator–prey relationships. Understanding the relative role of top‐down versus bottom‐up drivers is of particular interest in understanding ecosystem dynamics. Our goal was to explore predator–prey relationships in a boreal ecosystem in interior Alaska through the use of multispecies long‐term monitoring data. We used 29 years of field data and a dynamic multistate site occupancy modeling approach to explore the trophic relationships between an apex predator, the golden eagle, and cyclic populations of the two primary prey species available to eagles early in the breeding season, snowshoe hare and willow ptarmigan. We found that golden eagle reproductive success was reliant on prey numbers, but also responded prior to changes in the phase of the snowshoe hare population cycle and failed to respond to variation in hare cycle amplitude. There was no lagged response to ptarmigan populations, and ptarmigan populations recovered quickly from the low phase. Together, these results suggested that eagle reproduction is largely driven by bottom‐up processes, with little evidence of top‐down control of either ptarmigan or hare populations. Although the relationship between golden eagle reproductive success and prey abundance had been previously established, here we established prey populations are likely driving eagle dynamics through bottom‐up processes. The key to this insight was our focus on golden eagle reproductive parameters rather than overall abundance. Although our inference is limited to the golden eagle–hare–ptarmigan relationships we studied, our results suggest caution in interpreting predator–prey abundance patterns among other species as strong evidence for top‐down control.

## 1 INTRODUCTION

Identifying the drivers of population dynamics is a central goal in the field of population ecology (Williams, Nichols, & Conroy, 2002), particularly in the context of predator–prey dynamics. Population dynamics are influenced by biotic and abiotic factors, although the role of predation in driving prey populations has received particular attention (e.g., Arditi & Ginzburg, 1989; Holt, 1977; Sinclair, Mduma, & Brashares, 2003). Predators limit prey populations through top‐down mechanisms in many systems (Baum & Worm, 2009; Frank, Petrie, Choi, & Leggett, 2005; McLaren & Peterson, 1994; Therrien, Gauthier, Korpimaki, & Bety, 2014), although bottom‐up limitation can also play a role (Frederiksen, Edwards, Richardson, Halliday, & Wanless, 2006; Schmidt et al., 2017; Ware & Thomson, 2005). Identifying the relative roles of top‐down versus bottom‐up forces is crucial in understanding ecosystem dynamics and the population dynamics of both predators and their prey.

Boreal ecosystems are generally less productive and structurally simpler than those at lower latitudes, providing an opportunity to investigate predator–prey relationships in the context of fewer confounding trophic relationships (Krebs, Boonstra, Boutin, & Sinclair, 2001; Krebs, Boutin, & Boonstra, 2001). The dominant prey species in the boreal forest across much of North America is the snowshoe hare [*Lepus americanus*], which exhibits regular 9–11 year population cycles (Hodges et al., 2001; Keith, Cary, Rongstad, & Brittingham, 1984; Keith & Windberg, 1978). The hare cycle is closely tracked by their primary predator, the Canada lynx [*Lynx canadensis*] (Krebs, Boonstra, et al., 2014; O'Donoghue et al., 2001). The willow ptarmigan [*Lagopus lagopus*] is another important prey species whose population cycles in phase with snowshoe hare populations in many boreal systems (Boutin et al., 1995; McIntyre & Schmidt, 2012). Predation is the proximate cause of a majority of mortalities in hares (Krebs, 2011; Krebs, Boutin, et al., 2001; Krebs, Boonstra, et al., 2001), and although less well understood, ptarmigan as well (Martin, Doyle, Hannon, & Mueller, 2001; Nielsen, 1999; Sandercock, Nilsen, Broseth, & Pedersen, 2011; Smith & Willegrand, 1999). While predators and prey populations often cycle together in the north, determining the causal factors for these patterns remains one of the central questions in ecology (Boonstra & Krebs, 2012; Krebs, 2011, 2013; Krebs et al., 1995; Krebs, Boutin, et al., 2001; Krebs, Boonstra, et al., 2001; Stenseth, 1999).

Raptors represent one component of a suite of predators in boreal systems (Boutin et al., 1995; Krebs, Boutin, et al., 2001; Krebs, Boonstra, et al., 2001), although they often receive less attention than their mammalian counterparts. Due to their territorial nature and life history characteristics (i.e., use of readily observable nests for breeding), assessments of reproduction are much more feasible for some raptors than for many free‐ranging mammals. Detailed information on reproductive dynamics relative to important prey resources provides unique opportunities to investigate the trophic relationships between raptors and their prey as part of the larger ecosystem.

Much of the research on predator–prey dynamics focuses on changes in the overall abundance of predators and their prey, although raptor research has revealed that reproductive effort and success are often linked to fluctuations in prey resources (e.g., great‐horned owls [*Bubo virginianus*], Rohner, Doyle, & Smith, 2001; gyrfalcons [*Falco rusticolus*], Nielsen, 2011; Tengmalm's owls [*Aegolius funereus*], Korpimaki, 1992; golden eagles [*Aquila chsaetos*], Steenhof, Kochert, & McDonald, 1997; McIntyre & Schmidt, 2012). These findings suggest study of the reproductive dynamics of raptors relative to their prey might reveal some of the mechanisms behind these relationships.

We monitored a breeding population of golden eagles and their primary spring prey, snowshoe hare (hare), and willow ptarmigan (ptarmigan), in Denali National Park and Preserve (Denali) over a 29‐year period (1988–2016). This long‐term dataset provides a unique opportunity to assess the relationships between this apex predator and its primary spring prey. Eagles in this population are migratory, wintering at more southern latitudes and returning to their breeding grounds in late winter or early spring (Kochert, Steenhof, McIntyre, & Craig, 2002; McIntyre, Douglas, & Collopy, 2008). During the nest initiation period (late March, early April), snowshoe hare and willow ptarmigan are the only golden eagle prey species that are functionally available and widely distributed on the landscape. Accordingly, these two species make up the majority of the diet of golden eagles in Denali during the prelaying and early incubation periods. While arctic ground squirrels (*Spermophilus parryii*) constitute a major portion of the diet of nesting eagles in Denali, they do not become readily available until well after most eagles have completed their clutches (McIntyre & Schmidt, 2012). Correspondingly, hare and ptarmigan were identified as important correlates of eagle reproductive success (McIntyre & Adams, 1999; McIntyre & Schmidt, 2012); however, the mechanisms behind this relationship have not been assessed. To assess whether eagles were more likely limiting (i.e., top‐down) or limited by (i.e., bottom‐up) their spring prey populations in Denali, we used a dynamic multistate occupancy modeling framework (MacKenzie, Nichols, Seamans, & Gutiérrez, 2009) to: 1) quantify the relationship between eagle reproductive dynamics and hare and ptarmigan abundance, 2) assess whether eagle reproductive parameters responded to variation in prey cycle amplitude, and 3) assess whether eagle reproductive metrics simply tracked prey abundance or changed prior to shifts in prey resources (i.e., lagged effects).

## 2 METHODS

### 2.1 Study area

**Figure 1:**
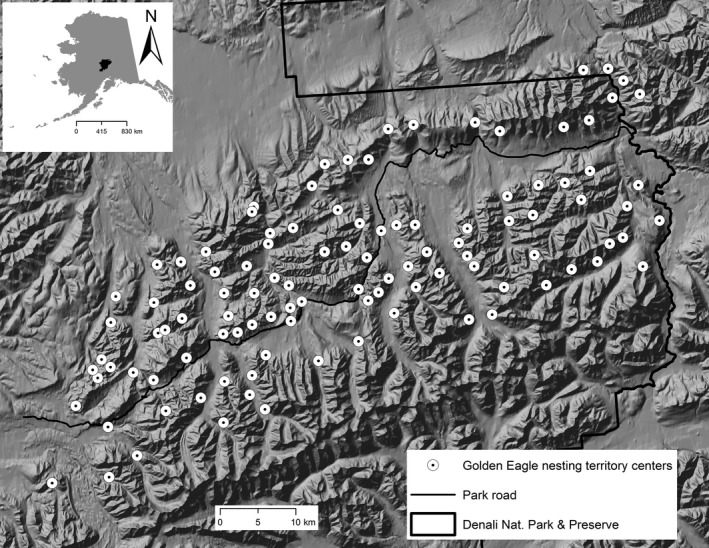
Location of Denali National Park and Preserve within the state of Alaska (inset) and the approximate locations of the golden eagle territories monitored from 1988 to 2016

Our study area encompassed 2,522 km^2^ in the northern foothills of the Alaska Range within Denali in interior Alaska, USA (Figure 1). The study area encompasses boreal–montane ecosystems including conifer and mixed forests in the lowlands ranging upward into subalpine shrublands and then through an alpine tundra zone into barren rock and ice in the highest elevations (Roland, Schmidt, & Nicklen, 2013). The topography ranges from rugged mountainous terrain to broad glacial valleys, as well as upland areas. Elevations ranged from ~400 m to 1,400 m. The area experiences long, cold, dry winters and short, warm summers. The average annual temperature is −3°C, with an average high of 20°C and an average low of −22°C (Shulski & Wendler, 2007). Average annual precipitation is 38.1 cm (Shulski & Wendler, 2007). Annual snowfall averages 206 cm, which is usually concentrated in the months of November and December (Sousanes, 2008).

### 2.2 Field methods

From 1988 to 2016, we collected data on the occupancy state of nesting territories using two standardized aerial and ground surveys (see McIntyre & Schmidt, 2012). The first survey was conducted in late April and early May after clutch completion but before hatching. We attempted to observe all known nests and actively searched for unknown or new nests within each territory during the first survey. Each territory was observed in one of four mutually exclusive occupancy states: unoccupied, occupied (territorial pair present with no evidence of reproductive activity), nesting attempted (egg laying without success), or successful reproduction (≥1 fledgling observed). When the occupancy state of a territory was inconclusive, we conducted additional aerial or ground surveys to assure assignment to the appropriate occupancy state. We completed the second survey in mid‐July to late July to revisit all nests that were initially assigned to the “nesting attempted” state to document nest success (McIntyre & Schmidt, 2012). The second author conducted surveys of up to 90 of the 103 known territories by small helicopter and foot travel annually throughout the study period (Figure 1). Further details regarding field methods can be found in McIntyre and Schmidt (2012).

We indexed annual hare and ptarmigan abundance from 1988 to 2016 as the average number of adults of each species observed per day during routine fieldwork conducted throughout the study area from mid‐April to late June. Ptarmigan indices were based primarily on observations of territorial males. All observations were made and recorded by the same individual (C. McIntyre) using a standardized approach for the entire period (see McIntyre & Adams, 1999; McIntyre & Schmidt, 2012). While the annual indices could not be converted to true abundance, we are confident that this measure accurately represented relative abundance between years and the frequency and relative amplitude of the hare and ptarmigan cycles in Denali (see Krebs et al., 2013). The hare index was in general agreement with an assessment of the hare cycle in an adjacent area (Arthur & Prugh, 2010), further supporting our assertion that our data accurately represented the relative abundance of these species in Denali.

### 2.3 Statistical analysis

We used dynamic multistate site occupancy models (e.g., MacKenzie et al., 2009; Martin et al., 2009) to assess the detailed occupancy dynamics of eagle territories relative to variation in hare and ptarmigan indices. Detection probability for each visit was quite high, and multiple visits to individual territories ensured that identification of occupancy state was ≈1.0 (Martin et al., 2009; McIntyre & Schmidt, 2012). Therefore, for the purposes of analysis, we assumed that errors in the classification of occupancy state did not occur.

We used a model parameterization similar to that presented by Kéry and Schaub (2012) and Schmidt, Flamme, and Walker (2014), extended to include four mutually exclusive occupancy states, $\Psi^{\left[k\right]}:$ [1] unoccupied, [2] occupied, [3] nesting attempted, and [4] successful reproduction. Our notation generally follows that of Schmidt et al. (2014) where the probability of a territory occurring in each of the four potential states in year_t_ can be written as:

$$\Psi^{\left[1\right]}=1-\left(\phi^{\left[2\right]}+\gamma^{\left[1\right]\left[2\right]}+\gamma^{\left[3\right]\left[2\right]}+\gamma^{\left[4\right]\left[2\right]}\right)$$

$$\begin{array}{cc} \Psi^{\left[2\right]}= & \left(\phi^{\left[2\right]}+\gamma^{\left[1\right]\left[2\right]}+\gamma^{\left[3\right]\left[2\right]}+\gamma^{\left[4\right]\left[2\right]}\right)*\left(1-\left(\phi^{\left[3\right]}+\gamma^{\left[1\right]\left[3\right]}+\gamma^{\left[2\right]\left[3\right]}+\gamma^{\left[4\right]\left[3\right]}\right)\right)\\ & *\left(1-\left(\phi^{\left[4\right]}+\gamma^{\left[1\right]\left[4\right]}+\gamma^{\left[2\right]\left[4\right]}+\gamma^{\left[3\right]\left[4\right]}\right)\right) \end{array}$$

$$\begin{array}{cc} \Psi^{\left[3\right]}= & \left(\phi^{\left[2\right]}+\gamma^{\left[1\right]\left[2\right]}+\gamma^{\left[3\right]\left[2\right]}+\gamma^{\left[4\right]\left[2\right]}\right)*\left(\phi^{\left[3\right]}+\gamma^{\left[1\right]\left[3\right]}+\gamma^{\left[2\right]\left[3\right]}+\gamma^{\left[4\right]\left[3\right]}\right)\\ & *\left(1-\left(\phi^{\left[4\right]}+\gamma^{\left[1\right]\left[4\right]}+\gamma^{\left[2\right]\left[4\right]}+\gamma^{\left[3\right]\left[4\right]}\right)\right) \end{array}$$

$$\begin{array}{cc} \Psi^{\left[4\right]}= & \left(\phi^{\left[2\right]}+\gamma^{\left[1\right]\left[2\right]}+\gamma^{\left[3\right]\left[2\right]}+\gamma^{\left[4\right]\left[2\right]}\right)*\left(\phi^{\left[3\right]}+\gamma^{\left[1\right]\left[3\right]}+\gamma^{\left[2\right]\left[3\right]}+\gamma^{\left[4\right]\left[3\right]}\right)\\ & *\left(\phi^{\left[4\right]}+\gamma^{\left[1\right]\left[4\right]}+\gamma^{\left[2\right]\left[4\right]}+\gamma^{\left[3\right]\left[4\right]}\right) \end{array}$$

where ϕ represents the probability of remaining in the same state as in year_t−1._ The probability of transitioning from one state to another between year_t−1_ and year_t_ is γ. The first superscript on γ indicates the true state in year_t−1_, and the second represents the true state in year_t_. Further details on model structure and notation can be found in Kéry and Schaub (2012) and Schmidt et al. (2014).

Each ϕ and γ was modeled as a function of covariates. These submodels can be written as:

$$\begin{array}{cc} logit\left(\phi^{\left[k\right]}\right)= & \beta_{0\phi }^{\left[k\right]}+\beta_{1\phi }^{\left[k\right]}hares_{t}+\beta_{2\phi }^{\left[k\right]}hares_{t-1}+\beta_{3\phi }^{\left[k\right]}ptarm_{t}+\beta_{4\phi }^{\left[k\right]}ptarm_{t-1}\\ & +\beta_{5\phi }^{\left[k\right]}\beta_{\phi 5}year_{t} \end{array}$$

$$\begin{array}{cc} logit\left(\gamma^{\left[k^{*}\right]\left[k\right]}\right)= & \beta_{0\gamma }^{\left[k^{*}\right]\left[k\right]}+\beta_{1\gamma }^{\left[k^{*}\right]\left[k\right]}hares_{t}+\beta_{2\gamma }^{\left[k^{*}\right]\left[k\right]}hares_{t-1}+\beta_{3\gamma }^{\left[k^{*}\right]\left[k\right]}ptarm_{t}\\ & +\beta_{4\gamma }^{\left[k^{*}\right]\left[k\right]}ptarm_{t-1}+\beta_{5\gamma }^{\left[k^{*}\right]\left[k\right]}\beta_{\gamma 5}year_{t} \end{array}$$

where *k* ^*^ represents the true state in year_t−1_. We began by assuming that the relative numbers of hares and ptarmigan (ptarm), as well as trends through time (year), would be the predominant drivers of occupancy dynamics based on the work of McIntyre and Schmidt (2012). However, in contrast to previous work, we also considered the abundance of both prey species in year_t−1_ relative to the reproductive state of each territory in year_t_. This allowed us to assess whether patterns in golden eagle occupancy states tracked prey populations directly or prior years’ prey numbers as well. We fit models with and without the lagged prey covariates and trend and used model selection procedures based on DIC to select the most parsimonious model. Models were fit using program R (v. 2.14.2, R Development Core Team 2012) and OpenBUGS (Thomas, O'Hara, Ligges, & Sturtz, 2006). We ran two independent Markov chains for 10,000 iterations each, discarding the first 7,000 as burn‐in. The remaining 3,000 were retained for inference.

## 3 RESULTS

**Figure 2:**
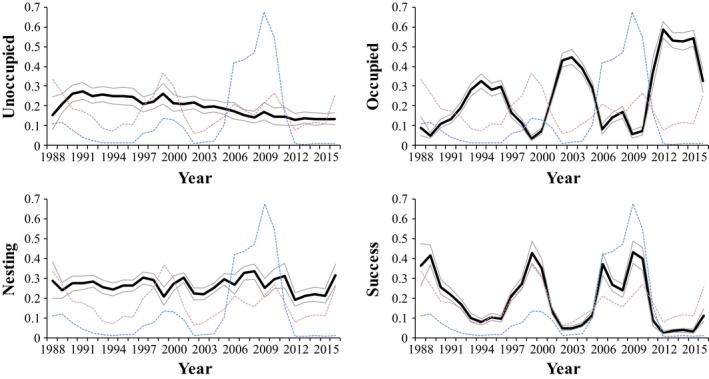
Annual proportion of each of the 103 monitored GOEA territories in one of four occupancy states: unoccupied, occupied, nesting attempted, and successful reproduction in Denali National Park and Preserve, Alaska, USA. Annual hare (blue) and willow ptarmigan (red) indices (scaled for presentation) are also included for reference

The full model was the next best model compared to our top model (ΔDIC = 13), indicating little model selection uncertainty relative to the current and lagged effects of these two prey species on occupancy state. The probability of eagles successfully raising fledglings was positively related to the indices of hare and ptarmigan abundance (Table 1, Figure 2), as we expected. Territories with attempted or successful nests in year_t−1_ were more likely to remain in the same state in year_t_ when hare and ptarmigan numbers were high in year_t_. For example, the positive value of $\beta_{1\gamma }^{\left[2\right]\left[3\right]}$ indicated that the probability of transitioning from occupied in year_t−1_ to attempted nesting in year_t_ was higher in years when the hare index was higher (Table 1). Territories were also more likely to transition from occupied to attempted or successful nesting when hare and ptarmigan numbers were higher in year_t_. However, there was a negative relationship between hare abundance in year_t−1_ and the probability of a territory transitioning from an occupied state to either attempted nesting ($\beta_{2\gamma }^{\left[2\right]\left[3\right]}$ = −0.39) or successful reproduction in year_t_ ($\beta_{2\gamma }^{\left[2\right]\left[4\right]}$ = −0.52). This indicated that the probability of golden eagle nesting attempts and successful reproduction did not track current hare numbers alone, but began to slow prior to each peak in the hare cycle and increase more rapidly after the hare cycle reached its nadir than would be expected based on hare and ptarmigan numbers in year_t_ alone. There was no corresponding lagged response to ptarmigan numbers. Trends in occupancy states over time indicated that occupancy without reproduction increased, while nesting attempts and successful reproduction decreased after accounting for prey abundance. Interestingly, the proportion of territories with attempted nesting did not vary appreciably in relation to prey abundance, although the probability of nesting declined slowly through time (Table 1, Figure 2). In addition, although the third hare peak was ~fourfold larger than the previous two, golden eagle reproductive parameters did not show a corresponding response (Figure 2). In contrast, both attempted nesting and successful reproduction actually declined through time despite the large increase in the amplitude of the hare cycle during the latter part of the study. Overall, golden eagle reproductive effort (i.e., nesting attempted and successful reproduction) declined through time, while territory occupancy increased. The lack of a direct response to the increased amplitude of the third hare peak indicated that eagles failed to fully respond to large increases in prey resources.

**Table 1:** Estimates for coefficients in the top dynamic multistate model representing golden eagle occupancy dynamics

Parameter	Mean	95% CI
$\beta_{0\phi }^{\left[2\right]}$	**2.58**	**(2.30, 2.88)**
$\beta_{0\phi }^{\left[3\right]}$	**0.51**	**(0.28, 0.77)**
$\beta_{0\phi }^{\left[4\right]}$	**−0.67**	**(−0.86, −0.47)**
$\beta_{0\gamma }^{\left[1\right]\left[2\right]}$	**−1.02**	**(−1.25, −0.81)**
$\beta_{0\gamma }^{\left[3\right]\left[2\right]}$	**3.14**	**(2.58, 3.93)**
$\beta_{0\gamma }^{\left[4\right]\left[2\right]}$	**3.26**	**(2.80, 3.74)**
$\beta_{0\gamma }^{\left[1\right]\left[3\right]}$	**0.69**	**(0.25, 1.13)**
$\beta_{0\gamma }^{\left[2\right]\left[3\right]}$	0.12	(**−**0.07, 0.31)
$\beta_{0\gamma }^{\left[4\right]\left[3\right]}$	**0.42**	**(0.24, 0.60)**
$\beta_{0\gamma }^{\left[1\right]\left[4\right]}$	**−0.46**	**(−0.91, −0.04)**
$\beta_{0\gamma }^{\left[2\right]\left[4\right]}$	**−0.81**	**(−0.99, −0.63)**
$\beta_{0\gamma }^{\left[3\right]\left[4\right]}$	**−0.57**	**(−0.83, −0.35)**
$\beta_{1\phi }^{\left[2\right]}$	**−**0.35	(**−**0.85, 0.14)
$\beta_{1\phi }^{\left[3\right]}$	0.35	(**−**0.05, 0.71)
$\beta_{1\phi }^{\left[4\right]}$	**0.56**	**(0.31, 0.84)**
$\beta_{1\gamma }^{\left[1\right]\left[2\right]}$	**−**0.01	(**−**0.39, 0.38)
$\beta_{1\gamma }^{\left[3\right]\left[2\right]}$	0.84	(**−**0.46, 2.49)
$\beta_{1\gamma }^{\left[4\right]\left[2\right]}$	0.52	(**−**0.06, 1.14)
$\beta_{1\gamma }^{\left[1\right]\left[3\right]}$	0.31	(**−**0.42, 1.12)
$\beta_{1\gamma }^{\left[2\right]\left[3\right]}$	**0.74**	**(0.44, 1.05)**
$\beta_{1\gamma }^{\left[4\right]\left[3\right]}$	**0.41**	**(0.14, 0.66)**
$\beta_{1\gamma }^{\left[1\right]\left[4\right]}$	0.37	(**−**0.29, 1.03)
$\beta_{1\gamma }^{\left[2\right]\left[4\right]}$	**0.80**	**(0.49, 1.13)**
$\beta_{1\gamma }^{\left[3\right]\left[4\right]}$	**0.41**	**(0.08, 0.78)**
$\beta_{2\phi }^{\left[2\right]}$	**−**0.15	(**−**0.63, 0.39)
$\beta_{2\phi }^{\left[3\right]}$	**−**0.03	(**−**0.40, 0.41)
$\beta_{2\phi }^{\left[4\right]}$	**−**0.11	(**−**0.39, 0.17)
$\beta_{2\gamma }^{\left[1\right]\left[2\right]}$	0.13	(**−**0.27, 0.53)
$\beta_{2\gamma }^{\left[3\right]\left[2\right]}$	0.49	(**−**0.66, 1.81)
$\beta_{2\gamma }^{\left[4\right]\left[2\right]}$	**−**0.59	(**−**1.11, 0.00)
$\beta_{2\gamma }^{\left[1\right]\left[3\right]}$	**−**0.17	(**−**0.84, 0.60)
$\beta_{2\gamma }^{\left[2\right]\left[3\right]}$	**−0.39**	**(−0.68, −0.09)**
$\beta_{2\gamma }^{\left[4\right]\left[3\right]}$	0.04	(**−**0.21, 0.31)
$\beta_{2\gamma }^{\left[1\right]\left[4\right]}$	0.07	(**−**0.58, 0.71)
$\beta_{2\gamma }^{\left[2\right]\left[4\right]}$	**−0.52**	**(−0.89, −0.18)**
$\beta_{2\gamma }^{\left[3\right]\left[4\right]}$	**−**0.06	(**−**0.43, 0.27)
$\beta_{3\phi }^{\left[2\right]}$	0.29	(**−**0.06, 0.68)
$\beta_{3\phi }^{\left[3\right]}$	**0.47**	**(0.20, 0.77)**
$\beta_{3\phi }^{\left[4\right]}$	**0.58**	**(0.38, 0.79)**
$\beta_{3\gamma }^{\left[1\right]\left[2\right]}$	**−**0.02	(**−**0.26, 0.23)
$\beta_{3\gamma }^{\left[3\right]\left[2\right]}$	**−**0.44	(**−**0.97, 0.08)
$\beta_{3\gamma }^{\left[4\right]\left[2\right]}$	**−**0.30	(**−**0.73, 0.13)
$\beta_{3\gamma }^{\left[1\right]\left[3\right]}$	**0.90**	**(0.33, 1.55)**
$\beta_{3\gamma }^{\left[2\right]\left[3\right]}$	**0.56**	**(0.37, 0.74)**
$\beta_{3\gamma }^{\left[4\right]\left[3\right]}$	**0.60**	**(0.38, 0.82)**
$\beta_{3\gamma }^{\left[1\right]\left[4\right]}$	**0.73**	**(0.25, 1.27)**
$\beta_{3\gamma }^{\left[2\right]\left[4\right]}$	**0.51**	**(0.34, 0.69)**
$\beta_{3\gamma }^{\left[3\right]\left[4\right]}$	**0.30**	**(0.01, 0.58)**
$\beta_{5\phi }^{\left[2\right]}$	**0.38**	**(0.06, 0.71)**
$\beta_{5\phi }^{\left[3\right]}$	**−0.41**	**(−0.70, −0.14)**
$\beta_{5\phi }^{\left[4\right]}$	**−0.47**	**(−0.72, −0.23)**
$\beta_{5\gamma }^{\left[1\right]\left[2\right]}$	0.16	(**−**0.10, 0.41)
$\beta_{5\gamma }^{\left[3\right]\left[2\right]}$	0.37	(**−**0.15, 0.86)
$\beta_{5\gamma }^{\left[4\right]\left[2\right]}$	0.45	(**−**0.06, 0.98)
$\beta_{5\gamma }^{\left[1\right]\left[3\right]}$	**−**0.19	(**−**0.67, 0.27)
$\beta_{5\gamma }^{\left[2\right]\left[3\right]}$	**−0.35**	**(−0.52, −0.19)**
$\beta_{5\gamma }^{\left[4\right]\left[3\right]}$	**−0.43**	**(−0.65, −0.20)**
$\beta_{5\gamma }^{\left[1\right]\left[4\right]}$	**−0.69**	**(−1.25, −0.16)**
$\beta_{5\gamma }^{\left[2\right]\left[4\right]}$	**−0.32**	**(−0.51, −0.14)**
$\beta_{5\gamma }^{\left[3\right]\left[4\right]}$	**−0.44**	**(−0.77, −0.16)**

Subscripts on model parameters, β, indicate effect of: intercept (0), hare index in year_t_ (1), hare index in year_t−1_ (2), ptarmigan index in year_t_ (3), and trend through time (5) on the probability of remaining in the same state between year_t_ and year_t−1_, ϕ, and the probability of transitioning among states between year_t_ and year_t−1_, γ. Superscripts in brackets represent true occupancy states: 1 = unoccupied, 2 = occupied, 3 = occupied with nesting, 4 = occupied with successful reproduction. All estimates are presented on the logit scale, and all covariates were scaled prior to analysis. Bolded rows indicate estimates with 95% credible intervals that do not overlap 0.

## 4 DISCUSSION

Long‐term datasets on the dynamics of naturally occurring populations of both predators and their primary prey within a single system are rare. Golden eagle population ecology in Denali has been studied extensively for almost 30 yrs (e.g., McIntyre & Adams, 1999; McIntyre & Collopy, 2006; McIntyre, Collopy, & Lindberg, 2006), and a direct relationship between eagle reproduction and prey abundance has been established (McIntyre & Schmidt, 2012). The unique findings in our current work were that eagle reproduction was also negatively related to hare production in the previous year and failed to fully respond to the 2006–2010 extreme hare high, in contrast to what would be predicted for a system under top‐down control. These findings indicated that eagle reproductive effort began to slow prior to the hare peak and then increase prior to the start of the recovery of the hare population. In addition, eagle reproductive metrics quickly began to recover in apparent response to the increase phase of the ptarmigan cycle, without “controlling” the ptarmigan population at low levels. Our results illustrate that eagle reproductive output in Denali (i.e., fledgling production) is largely controlled by bottom‐up forces rather than predation by eagles acting to limit hare and ptarmigan populations through top‐down mechanisms (e.g., White, 2013).

The boreal forest ecosystem in North America is dominated by the hare–lynx cycle which is generally thought to be driven primarily by lynx predation (e.g., Krebs, Boonstra, et al., 2001; Krebs, Boutin, et al., 2001; Krebs, Boonstra, et al., 2014). Top‐down forces may regulate prey densities in many systems (Fauteux, Gauthier, & Berteaux, 2016; Krebs, Boonstra, et al., 2001; Krebs, Boutin, et al., 2001; Sinclair et al., 2000); however, much of the existing predator–prey literature from this region is focused on the relationship between predator and prey abundance (but see Brommer, Pietiainen, & Kolunen, 2002; Millon & Bretagnolle, 2008) rather than assessments of the individual components of population dynamics (e.g., survival or reproduction). Additionally, data are often limited to coarse, indirect measures of abundance such as fur harvest records (Stenseth et al., 1998; Stenseth, Chan, et al., 1999) or track counts (Korpimaki, Norrdahl, & Rinta‐Jaskari, 1991; Krebs, Boonstra, et al., 2014; Krebs, Bryant, et al., 2014; O'Donoghue, Boutin, Krebs, & Hofer, 1997; O'Donoghue et al., 2001) containing little or no information on demographic rates. We argue that more detailed assessments of key demographic rates themselves may reveal apparently contrasting relationships between the vital rates of predators and their prey. Our results support this argument by providing evidence that an apex predator in our subarctic system, the golden eagle, is limited by bottom‐up processes through variation in prey resources. This finding suggests that further assessments of survival and reproductive rates in other predator species could reveal similar patterns.

Further investigation into demographic rates is needed for other predator populations in this system, particularly for lynx. Declines in predator abundance often lag behind those of prey (Krebs, Boonstra, et al., 2014; O'Donoghue et al., 1997, 1998, 2001; Stenseth et al., 1998) creating a pattern where predator populations remain high as prey decline, suggesting top‐down control of prey populations. Due to the coarse nature of the abundance indices available, it is plausible that demographic responses similar to those we observed in golden eagles may also occur in other predator species. We acknowledge that assessing individual vital rates in other important predators such as lynx is difficult; however, abundance‐based assessments of system dynamics may conceal important mechanistic relationships between predators and their prey. Although our inference is limited to the golden eagle–hare–ptarmigan relationships we studied, our results at least suggest caution in interpreting predator–prey abundance patterns among other species as strong evidence for top‐down control.

Strong associations between raptors and their prey have been observed in a variety of systems (e.g., Hoy, Millon, Petty, Whitfield, & Lambin, 2016; Resano‐Mayor et al., 2016; Salamolard, Butet, Leroux, & Bretagnolle, 2000), and although raptors may exhibit top‐down control (e.g., Thirgood, Redpath, Rothery, & Aebischer, 2000; Thirgood, Redpath, Haydon, et al., 2000), bottom‐up control has been identified in many raptor‐dominated systems. For example, reproduction in goshawk populations in Finland is limited by bottom‐up processes through heterogeneity in habitat composition and prey density (Byholm, Nikula, Kentta, & Taivalmaki, 2007). Furthermore, lifetime reproductive success in Tengmalm's owls is directly related to prey abundance (Korpimaki, 1992). Although we did not measure hare reproductive rates directly, hare productivity generally declines as the cycle nears the peak and begins to increase near the end of the low period (Krebs, Boonstra, et al., 2014), suggesting that golden eagles in Denali may be responding to the juvenile hare component of the population. It is also plausible that declines in reproductive output in hares may be related to density and/or food resources, further suggesting bottom‐up drivers. However, rigorous assessments of these hypotheses are certainly required before such linkages can be established.

Golden eagles have several life history characteristics that differ from those of other apex predators in our study area, potentially focusing the effects of prey on specific vital rates such as reproduction. First, although golden eagles may be classified as generalist predators in the sense that they utilize a variety of prey species throughout the year (Kochert et al., 2002; Watson, 2010), they specialize on hare and ptarmigan during the early part of the breeding season in Denali. Very few territories in our study area remained or transitioned to an unoccupied state, regardless of variation in prey abundance, suggesting that food resources in Denali are generally sufficient to maintain adult eagles (i.e., no decrease in survival), while eagle reproduction is limited by the abundance of primary prey. In contrast, both survival and recruitment rates for lynx, a nonmigratory species, vary dramatically depending on the phase of the hare cycle (Poole, 1994). The migratory nature of Denali's eagles may mitigate the effects of limited prey resources on adult survival, suggesting that the cost of migration may be mitigated by avoiding periods when prey availability may be further limited (i.e., winter). In contrast, resident specialist predators including lynx might be expected to experience large changes in multiple vital rates in response to variation in prey populations because they are unable to migrate to areas where prey are more available.

Overall, our work contributes to the basic understanding of predator–prey dynamics in boreal ecosystems. The dependence of golden eagle reproductive success on prey abundance has been well established (e.g., McIntyre & Adams, 1999; McIntyre & Schmidt, 2012; Steenhof et al., 1997), but our current work revealed the bottom‐up nature of this relationship in Denali. Although we focused on a single set of predator–prey relationships, our findings suggest that similar interactions may drive other predator–prey systems and that abundance‐based assessments might obscure the mechanisms driving changes in populations. We acknowledge that there are many other linkages that must be investigated before a full understanding of the dynamics of this system is realized; however, our work indicates that a more detailed investigation of vital rates (i.e., reproduction) may reveal unexpected relationships between prey resources and predator populations, possibly providing more conclusive evidence of the directional drivers (i.e., top‐down vs. bottom‐up) in a variety of predator–prey systems.

## DATA ACCESSIBILITY

The data used in this manuscript will be archived at: https://irma.nps.gov/DataStore/.

## AUTHOR CONTRIBUTION

CLM designed the field study and collected the data. JHS analyzed the data and wrote the manuscript. All authors contributed to the conceptual integration of datasets, discussed the results, and made substantive contributions to manuscript development and content.

## CONFLICT OF INTEREST

None declared.
